# Tailoring the Hydrogen Diffusion in Polycrystalline WO_3_ Thin Films by a p–n Heterojunction

**DOI:** 10.1021/acs.jpcc.5c04166

**Published:** 2025-10-03

**Authors:** Tim K. Hecker, Jan L. Dornseifer, Markus S. Friedrich, Martin Becker, Peter J. Klar

**Affiliations:** Institute of Experimental Physics I and Center for Materials Research, 9175 Justus Liebig University Giessen, Heinrich Buff Ring 16, 35392 Giessen, Germany

## Abstract

This study examines the lateral diffusion of hydrogen in tungsten trioxide (WO_3_) thin films with a nickel oxide (NiO) top layer. It focuses on the impact of the depletion region formed at the NiO/WO_3_ p–n heterojunction on the diffusion process. This depletion region influences diffusion by acting as a barrier to hydrogen movement. It effectively reduces the thickness in WO_3_ available for diffusion and increases the diffusion velocity due to the interplay with the concentration-dependent diffusion coefficient in polycrystalline WO_3_. Our in situ measurement technique allows for the detailed study of lateral hydrogen diffusion by inducing a concentration gradient in the layer plane. This method demonstrates by a direct comparison that diffusion is faster in the WO_3_/NiO layer structure compared to the pristine WO_3_ structure. This research demonstrates the technological potential of manipulating and tuning diffusion processes in electrochromic materials by incorporating them in layered structures and paves the way for more advanced applications.

## Graphical abstract

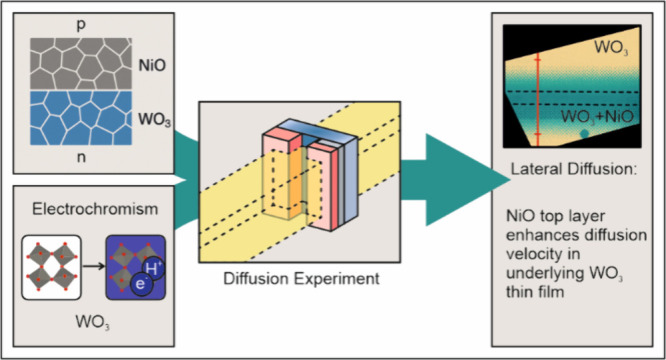

## 1 Introduction

Tungsten trioxide (WO_3_) has long been recognized for its electrochromic properties, which have been the subject of extensive research.^1–4^ The key to its optical property modifications lies in the reduction of the tungsten atoms by intercalation of small atomic species such as H or Li. For example, so-called tungsten bronzes H_*y*_WO_3_ are formed in case of hydrogen.^5^ Research has shown that in polycrystalline WO_3_ thin films, various crystal phases emerge in correlation with the concentration of intercalated hydrogen. The material undergoes a structural phase transition from a monoclinic phase to an orthorhombic phase at relatively low hydrogen concentrations.^6^ The crystal structure of pristine WO_3_ is in an orthorhombic or monoclinic phase. At approximately *y* = 0.1, the crystal lattice turns into a tetragonal phase,^6,7^ and, when the hydrogen concentration exceeds *y* = 0.5, it transforms into a cubic phase.^8^ These phase transitions are accompanied by significant changes in the materials properties regarding diffusion.

The diffusion of hydrogen in WO_3_ has been a subject of study for nearly half a century,^9^ producing a broad range of reported diffusion coefficients.^10–15^ Our group’s recent research has highlighted that the diffusion coefficient of hydrogen in WO_3_ thin films changes significantly during the structural phase transition from orthorhombic to tetragonal symmetry. Notably, in the phase with a higher symmetry, the diffusion coefficient increases by approximately 1 order of magnitude.^16^

While the bulk properties of WO_3_ and doped WO_3_ have been thoroughly investigated, the study of layered systems remains quite underexplored due to inherent challenges in measuring such configurations.^17–21^ Nevertheless, efforts are being made to incorporate semiconductor interfaces into devices for water splitting applications, which has led to an increased interest in understanding the underlying fundamentals.^22,23^ Our innovative and proven measurement approach focuses on lateral diffusion, enabling the examination of how interfaces to adjacent layers influence diffusion behavior in the WO_3_ layer plane. Our methodology introduced previously and refined in this paper allows for the in situ exploration of lateral hydrogen diffusion in thin films using a poly­(methyl methacrylate) (PMMA) structure with a stripe-like narrow gap for H^+^ intercalation.^24^

The measurement setup has been extended to analyze more complex systems, specifically a layered structure consisting of a WO_3_ layer partially covered by a nickel oxide (NiO) layer deposited by a sputter deposition process. NiO was selected because it is a semiconductor material exhibiting p-type conductivity,^26–31^ whereas WO_3_ is known for its n-type behavior.^27^ Hence, a NiO/WO_3_ interface will form a p–n junction.^27,32,33^ In addition, NiO shows good transmittance in the visible spectrum of light due to its high bandgap, an important feature for the transmission measurement. Also there are already quite some research papers addressing the p–n heterojunction between WO_3_ and NiO and studying their electronic properties.^27,32,33^ Also, devices for gas sensing applications utilizing the p–n heterojunction formed by WO_3_ and NiO are already being investigated.^34,35^ Hence we chose NiO as top layer, but in principle every p-type semiconductor should have a similar effect. The formation of the depletion regions due to the p–n heterojunction leads to a positively charged region located inside the n-type WO_3_ layer and a negatively charged region inside the p-type NiO layer. These depletion regions can expand up to the whole thickness of such thin films in some cases. In principle, NiO also shows anodic electrochromic behavior. However, this property does not play a role in our experiments as the voltages are not applied across the NiO layer and the incorporation of hydrogen is further inhibited by the formation of the p–n junction.

Our objective is to investigate the properties of the layer system and, in particular the effect of the p–n junction at the NiO/WO_3_ interface on electrochromism and diffusion behavior in the WO_3_ layer. We support our experimental findings by simulations that account for the heterogeneous hydrogen distribution across the layer thickness. This comprehensive approach provides new insights into the properties of layered electrochromic materials and shows the potential of leveraging electrochromic effects combined with interfaces.

## 2 Experimental Section

### 2.1 Sample Preparation

Formerly commercially available amorphous WO_3_ thin films from econtrol are etched with 1.938 M KOH (≥85% purity, from Roth GmbH + Co. KG) to uncover the underlying fluorine-doped tin oxide (FTO), which serves as contact during the measurement. The samples are then annealed in a Nabertherm furnace with air atmosphere with 100 °C/h up to 450 °C followed by 1 h at 450 °C. Subsequent to the crystallization a 150 nm thick NiO thin film is deposited with ion-beam sputter deposition on half of the sample using a technique developed in house^36^ using a nickel target (99% from Kurt J. Lesker Co. GmbH) and a gas mixture of 10 sccm O_2_ and 2 sccm argon with a substrate temperature of 500 °C. The previously uncovered FTO contact area is then covered with 6 ± 3 nm of Cr and 100 ± 15 nm of Au with a thermal evaporation system E12E4 from Edwards. The PMMA layer is applied by spin-coating with 150 μL of 4% PMMA in anisole solution (MicroChem Corp.) at 3000 rpm for 45 s on a Delta6 RC by SÜSS MicroTec AG. A XeDraw 2 electron beam lithography from XENOS in a JSM 7001F scanning electron microscope (SEM) from JEOL (acceleration voltage of 15 kV, beam current of 200 pA) is then used for the microstructuring process. The sample is then developed in isopropyl alcohol and deionized water with a ratio of 2:1.

### 2.2 Measurement Techniques for Sample Characterization

To measure the *U*–*I* characteristics of the sample we use a 4200-SCS semiconductor analyzer from Keithley. SEM imaging is carried out using a Zeiss Merlin HRSEM and energy dispersive X-ray spectroscopy (EDX) measurements with an Oxford Instruments 50 mm^2^X-max detector integrated to the SEM using Oxfords AZtec software for evaluation (acceleration voltage of 12 keV). Raman measurements are performed with an In-Via Raman spectrometer from Renishaw with a 633 nm laser. XRD measurements are carried out with a X’Pert Pro MRD from Pananalytical in 2θ-geometry. X-ray source is Cu Kα radiation with an energy of 1.546 eV from a copper anode. XPS measurements are conducted with a PHI Versaprobe II spectrometer with a source angle of 45°. Charge neutralization is done with Ar^+^ and e^–^ guns. Postcalibration is done with the C 1s signal. For depth profiling we use 120 s argon ion etching per step with an acceleration voltage of 1 kV. To determine the surface roughness of the sample a MultiMode 8-HR atomic force microscope from Bruker is used. The measurements are performed with the Bruker-specific noncontact mode ScanAsyst.

### 2.3 Electrochemical and Transmission Measurements

The setup is depicted in Figure 1b. Compared to our previous studies,^16^ it has now been extended to analyze the layered structure consisting of the WO_3_ layer partially covered by the NiO layer deposited by sputtering, shown in Figure 1a. In our sample structure, the edge of the NiO layer is aligned parallel to and near the PMMA-free stripe-like narrow gap. This configuration ensures that lateral diffusion processes can be studied simultaneously either side of the gap, i.e., below the PMMA film on the side of the gap where the PMMA film covers the WO_3_ layer directly and, on the other side, where the PMMA layer is coated on the NiO layer on top of WO_3_ layer.

**Figure 1:**
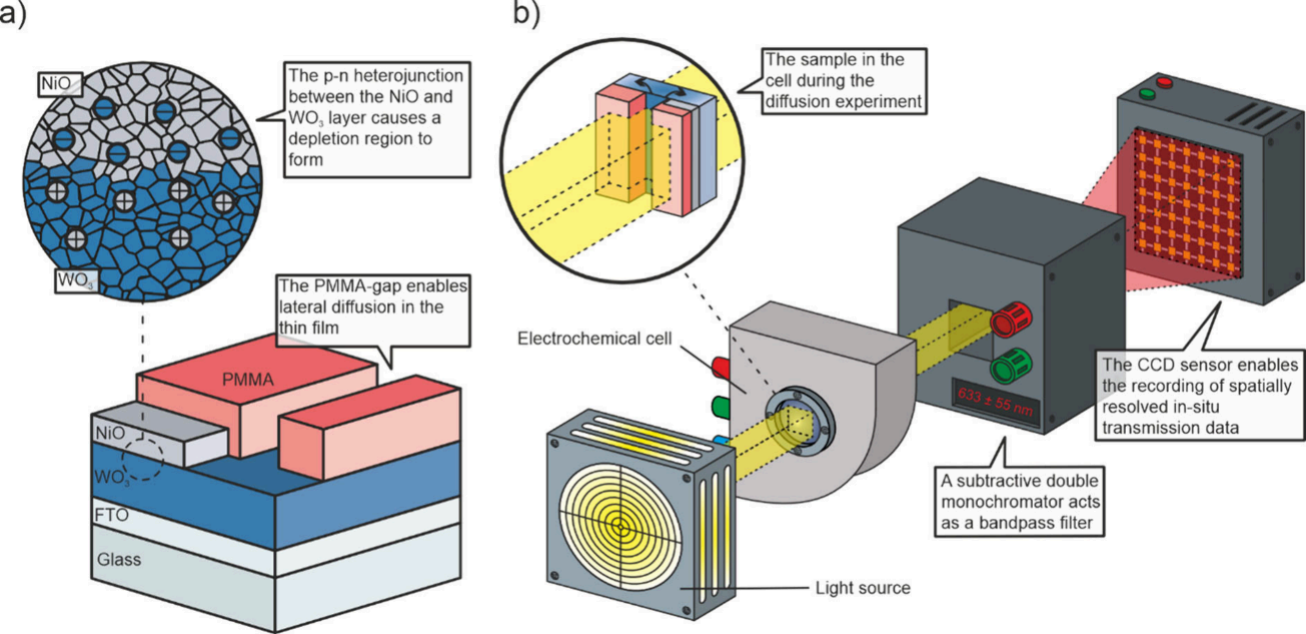
(a) Sample after the sputter deposition of NiO and the microstructuring of the PMMA film. The inset points out the p–n heterojunction at the NiO/WO_3_ intersection. (b) Measurement setup with light source, electrochemical cell, subtractive monochromator, and CCD sensor. The inset shows the sample orientation in the electrochemical cell during the measurement. The beam path is symbolic only and does not represent the actual path.

The actual electrochemical measurements are carried out in a cell developed in house^16,24^ using the microstructured sample as working electrode, platinum as counter electrode and a Ag/AgCl Driref-450 reference electrode from World Precision Instruments. As electrolyte 0.1 M eluent H_2_SO_4_ from Sigma-Aldrich is used. To apply the coloration and bleaching potentials of *E*_col_ = −0.2 V vs Ag/AgCl and *E*_blea_ = 0.8 V vs Ag/AgCl, respectively, a SP-150 potentiostat from Biologic is used. For the in situ 2D imaging the sample is illuminated with a halogen lamp. The transmitted light is then focused with a 10-fold magnification on a 6.0 mm entrance slit of a subtractive double spectrometer in Czerny–Turner geometry Spex 1680 b from Horiba Jobin Yvon GmbH, which basically acts as a bandpass filter and only transmits light in the range of λ = 633 ± 55 nm. The transmission data is then collected with a ICX258AL sensor from Sony in combination with a pco. 1400 CCD from PCO AG. The image size is 1392 × 1040 pixels with a pixel size of 6.45 × 6.45 μm^2^. To analyze pixel lines perpendicular to the PMMA gap a virtual instrument for the software LabVIEW from National Instruments Corp. is used.

## 3 Results and Discussion

### 3.1 Sample Preparation/Characterization

We utilize commercially available amorphous WO_3_ thin films with a thickness of about 480 nm, which are annealed at 450 °C for 1 h in air to induce crystallization. Postannealing of the films they yield polycrystalline properties while retaining a grain-like surface morphology (see Supporting Information Figure S1). Raman and X-ray diffraction (XRD) analysis confirm the coexistence of crystallites of monoclinic or orthorhombic crystal structure prior to intercalation, consistent with previous findings (see Figures S2 and S3). Subsequently, about 150 nm of NiO are deposited onto one-half of the surface of the WO_3_ film by ion-beam sputter-deposition at a substrate temperature of 500 °C. Surface morphology and crystal structure examinations after deposition indicate no alterations to the WO_3_ film. The grain-like morphology is maintained, with NiO forming small flakes that cover the WO_3_ grains (see Figure S1). XRD and Raman analysis confirm the identity and crystallinity of the NiO layer (see Figures S2 and S3). Furthermore, the analysis reveals that the crystalline properties of WO_3_ remain unchanged below the NiO layer. X-ray photoelectron spectroscopy (XPS) depth profiling of the layer structure (see Figure S4) reveals that tungsten is initially undetectable in the NiO layer but becomes noticeable with increasing depth close to the interface. After crossing the interface, nickel is no longer detected. This finding is indicative for a well-defined interface with minimal disorder between the layers. Its width is about 40 nm which is the same order of magnitude as the surface roughness of the WO_3_ layer after annealing (see Figure S5). Additionally, XPS yields a valence band offset between NiO and WO_3_ of Δ*E*_VB_ = 1.0 eV (see Figure S6). This result further corroborates the presence of distinct NiO and WO_3_ layers, with only minimal intermixing at the interface and an interface width given by the roughness of the WO_3_ layer.

These results are further substantiated by the SEM image of a cross-section of the sample, as depicted in Figure 2a. The NiO top layer can be clearly distinguished from the WO_3_ layer and is characterized by small rod-like crystallites that adapt to the surface morphology of the WO_3_, thereby retaining the grain-like surface texture. No evidence of intermixing with the underlying WO_3_ layer can be detected, but rather a clearly defined interface. In a similar manner, the WO_3_ layer is distinctly separated from the underlying FTO layer by a clear interface. These findings align with the results of the XPS depth profile, which revealed a well-defined interface and a disorder attributed to the surface morphology of the WO_3_ layer. The EDX measurements, depicted in Figure 2b, were performed on the same cross-section where the SEM image was recorded. In order to identify the various layers, we mapped the signals for the elements nickel, tungsten and tin. The mappings reveal three distinct layers and well-defined interfaces between them, thereby confirming quantitatively the visual impression of the SEM image. A supplementary analysis of an EDX line scan across the layers can be seen in Figures S7 and S8.

**Figure 2:**
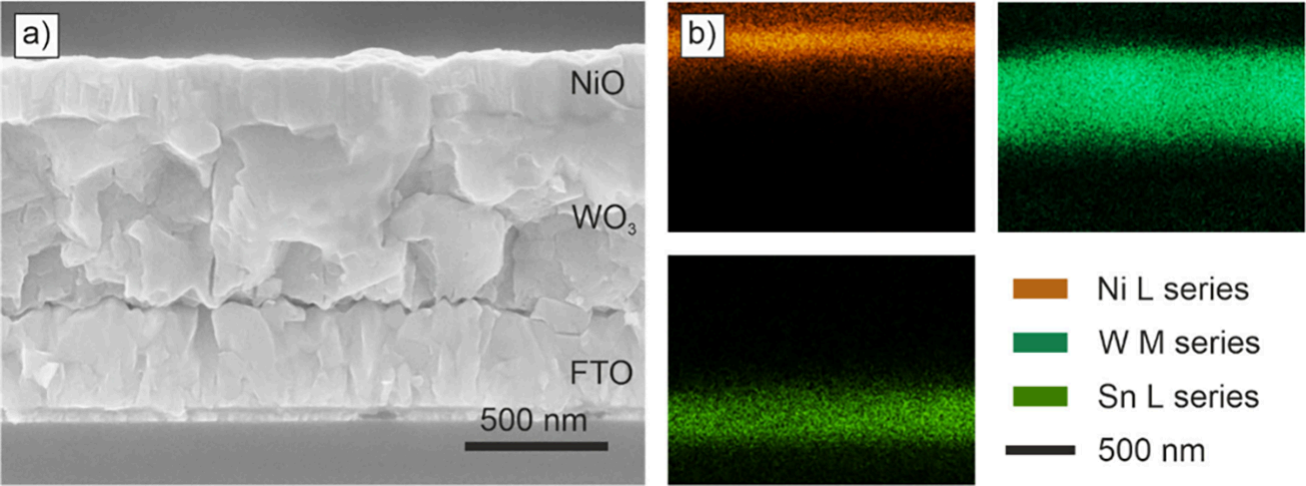
a) SEM-cross section of the sample showing the three different layers on top of the substrate. b) shows EDX elemental mappings of the sample cross section for Ni, W and Sn to identify the layers.

We also investigated the electronic properties of the layered WO_3_/NiO structure. By analyzing the *U*–*I* characteristic curve (Figure 3), we can identify diode-like behavior. This indicates the formation of a p–n junction with the associated depletion regions.^27,32,33^ However, the WO_3_/NiO diode structure is not ideal, i.e., the curve fitted to the *U*–*I* characteristic reveals a high ideality factor with the overall best fit being with an ideality factor of *n* = 21.27. We believe that this deviation from ideal behavior mainly arises due to two properties of the polycrystalline sputtered thin film structure. One is the roughness of the interface and the other is the presence of grains and the grain boundaries between them. In particular, the transport properties of the grain boundaries can differ considerably from those in the volume of the grains.^37,38^

**Figure 3:**
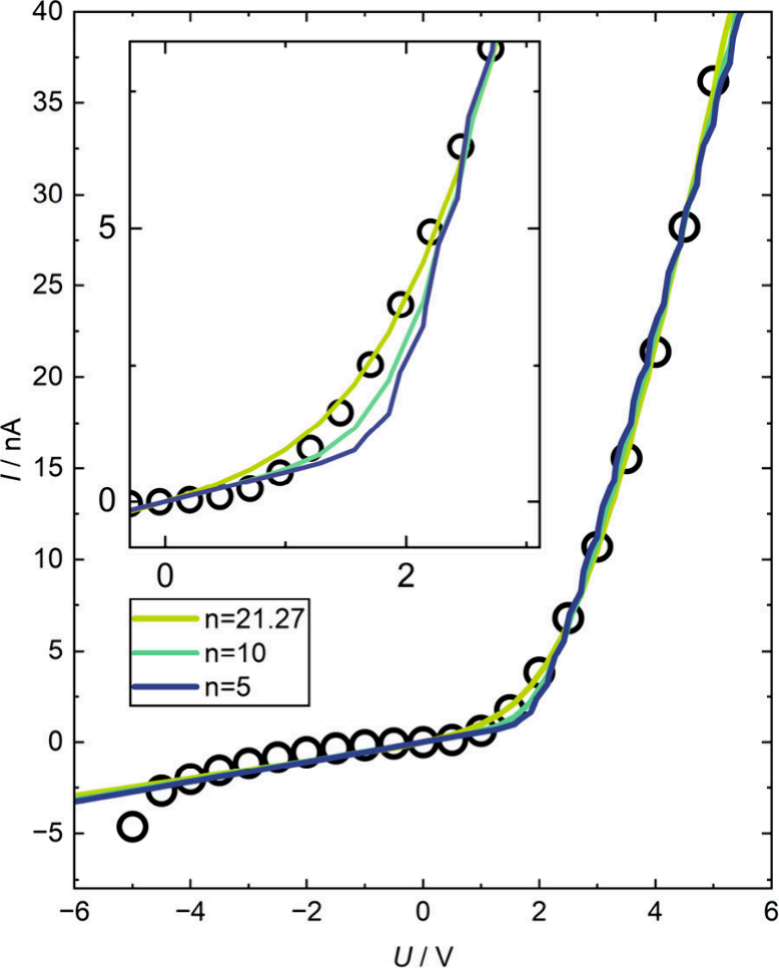
*U*–*I* characteristics of NiO/WO_3_ material system on FTO on glass substrate. Black circles represent every 100th data point (every 50th in the case of the inset). The colored lines represent fits with different ideality factors. Green is the best overall fit with an ideality factor of *n* = 21.27 and turquoise and blue represent fits with fixed ideality factors of 10 and 5, respectively. The inset shows a more detailed view of the differences between the three fits.

Understanding the width of this depletion region in WO_3_ is crucial for assessing its impact on hydrogen diffusion in the sample plane. Our estimates are based on the expression for the width of the depletion region in the n-type layer (here WO_3_) of an ideal p–n heterojunction with no applied voltage:

1 $$x_{n}=\sqrt{\frac{2}{e}\varepsilon_{0}\varepsilon_{p}\varepsilon_{n}\frac{N_{a}}{N_{d}}\frac{V_{\mathrm{bi}}}{\varepsilon_{p}N_{a}+\varepsilon_{n}N_{d}}}$$

and under an external voltage *V*, where the equation becomes

2 $$x_{n}\left(V\right)=\sqrt{\frac{2}{e}\varepsilon_{0}\varepsilon_{p}\varepsilon_{n}\frac{N_{a}}{N_{d}}\frac{V_{\mathrm{bi}}-V}{\varepsilon_{p}N_{a}+\varepsilon_{n}N_{d}}}$$

Here, *e* is the electron charge, *ε*_0_ is the vacuum permittivity, and *ε*_n_ and *ε*_p_ are the relative permittivities of WO_3_ and NiO, respectively. *N*_a_ and *N*_d_ are the acceptor and donor concentrations, while *V*_bi_ is the built-in potential of the junction, calculated to be approximately 1.08 V based on valence band offset measurements and other electronic properties (see Figure S6).

Literature provides a broad range of values for these parameters. For NiO, typical donor concentrations range from 10^18^ to 10^19^ cm^–3^,^31,39,40^ caused by nickel vacancies arising from excess oxygen during deposition.^28,31^ The acceptor concentration in WO_3_ lies between 10^15^ and 10^18^ cm^–3^.^32,41,42^ We estimate it to be around 2.6 × 10^17^ cm^–3^,^41^ based on a realistic oxygen vacancy concentration of about 10^–5^ per formula unit. The relative permittivity of NiO is in the low two-digit range.^33,43–46^ Literature data on WO_3_, on the other hand, exhibit a large spread in relative permittivity values.^42,47–49^ Using a typical value of the permittivity of WO_3_ of *ε*_n_ = 70,^49^ the expansion of the positively charged depletion region in the WO_3_ film, as predicted by eq 1, is about *x*_n_ = 170 nm. However, when the voltage perpendicular to the sample plane applied during the measurement is also considered as in eq 2, the width of the space charge zone is found to be even greater, about *x*_n_ = 200 nm. This value corresponds to about 40% of the thickness of the WO_3_ layer.

As absorption is not the sole process influencing the intensity of transmitted light, the reflection at the various interfaces was also taken into account. The incorporation of hydrogen and the subsequent coloration result in a change of the refractive index of WO_3_ from 2.2 to 1.2 at maximum hydrogen content (a level not reached in this experiment).^50^ We assume that no hydrogen diffuses into the NiO layer, thus its refractive index is constant. The same holds for the PMMA. Reflection takes place at an interface when there is a refractive index contrast between the layers forming the interface. Hydrogen intercalation into WO_3_ changes the refractive index contrast of two interfaces in the layer structure and thus their reflectivity and in turn their transmission behavior. The affected interfaces are the PMMA/WO_3_ and the WO_3_/NiO interface. Assuming a maximum effect in the change the refractive index of WO_3_ due to intercalation (i.e., disregarding the concentration gradient in the WO_3_ layer and the decreasing hydrogen concentration toward the NiO), the effect causes a change in the absorbance of at maximum of 0.09 (see eq S12). This value is much lower than the absorbance changes observed in the experiment of Δ*A* = 0.40 which are analyzed in terms of hydrogen induced coloration of the WO_3_ thin film.

### 3.2 Diffusion Experiment

For the lateral diffusion experiment almost the entire sample surface is covered with a thin PMMA layer to prevent direct hydrogen intercalation via the WO_3_ layer’s surface, with the exception of a microstructured stripe-like narrow gap where the WO_3_ surface is in direct contact with the electrolyte. The localized intercalation of hydrogen induces a concentration gradient in the plane of the WO_3_ thin film perpendicular to the microstructured narrow gap, thus facilitating lateral diffusion beneath the protective PMMA layer. This approach effectively extends the available diffusion path from a few hundred nanometres of the film’s thickness to a theoretically semi-infinite space, thereby enhancing both the measurement’s spatial and temporal resolution by several orders of magnitude. For these measurements, we monitor transmission within the 633 ± 55 nm spectral window spatially and temporally by imaging the sample surface with a subtractive double monochromator onto the chip of the CCD detector. Lambert–Beer’s law

3 $$A\left(x\right)=-\ln\left(\frac{I\left(x\right)}{I_{0}}\right)=\varepsilon c_{H}d\propto y\left(x\right)$$

applies,^25^ meaning that we can directly investigate the hydrogen concentration *y*(*x*) during the diffusion process by analyzing the transmission data.

The sample with the microstructured PMMA protective layer with its 50 μm wide stripe-like gap are mounted in the electrochemical cell for transmission measurements in such a way that the sample surface either side of the stripe-like narrow gap in the PMMA is imaged onto the CCD chip. Thus, a single measurement run yields simultaneously information about hydrogen diffusion in the WO_3_ layer covered by NiO with PMMA on top and in the same WO_3_ layer covered by PMMA only. In this way, measurement uncertainties can be eliminated as the comparison of the behavior can be made simultaneously on one and the same sample in one measurement run. Differences in diffusion behavior can therefore be directly attributed to the presence of the NiO on top of the WO_3_ since it is the only distinguishing factor between the two sides of the stripe-like narrow gap in the PMMA.^16,24^

Figure 4a shows the 2D CCD images of the transmission data *I*(*X*,*Y*) at the beginning of electrochemical coloration process (*t* = 0 s) and after 1 h of coloration (*t* = 3600 s). The PMMA free gap on the sample surface is indicated by the dashed black lines. Above the free gap is the area (region I) where the WO_3_ thin film is covered by the NiO layer with the PMMA on top of the latter. Below the free gap is the area (region II) where the WO_3_ thin film is covered by the PMMA only and no NiO layer is present. During coloration at a potential of *E*_we_vs Ag/AgCl = −0.2 V, only the area (i.e., the PMMA-free gap) where the electrolyte is in direct contact with the WO_3_ layer is initially colored. The hydrogen diffuses laterally in the WO_3_ thin film to either side of the free gap, i.e., into region I and II. The diffusion is driven by the concentration gradient perpendicular to the gap. On closer inspection, the diffusion fronts that form appear to have a different velocity in the region I and II as indicated by the purple ticks, which denote the same distance from the PMMA gap. The diffusion front in the WO_3_ covered with NiO (region I) has propagated 15 μm further away from the gap compared to that in the WO_3_ covered with PMMA only (region II).

**Figure 4:**
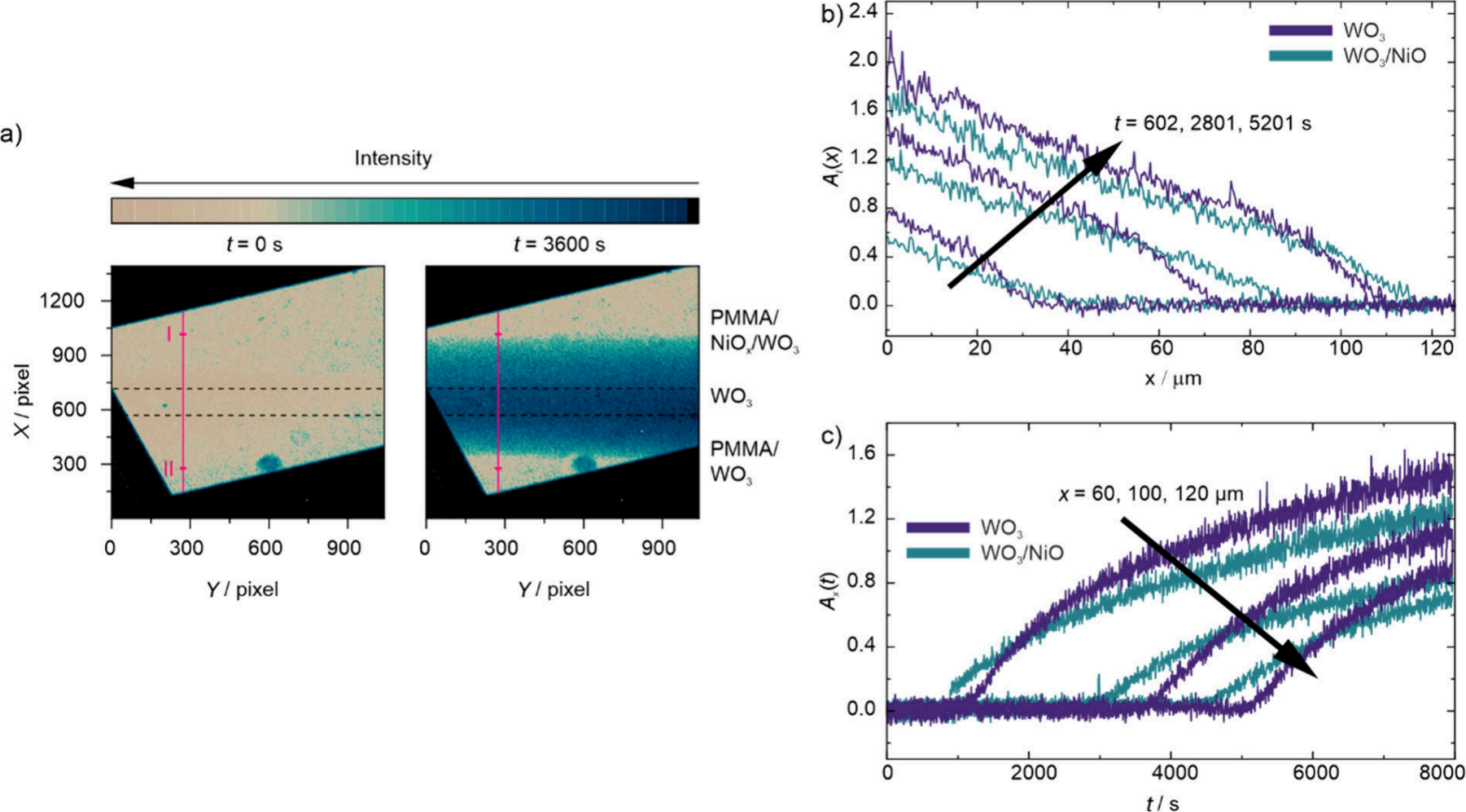
(a) 2D image as recorded by the CCD sensor at the start of the measurement and after 1 h; (b) absorbance/distance plot at three different times during the measurement; (c) absorbance/time plot for three different distances from the PMMA gap. For both plots purple represents the WO_3_ side directly covered by PMMA and blue the NiO/WO_3_ side covered by PMMA.

A more detailed interpretation of the data is possible by looking at 1D curves recorded at different times *t* into the coloration process at the single pixel column denoted by the purple line in Figure 4a, which is perpendicular to the PMMA slit. Each 1D curve *I*_*t*_(*X*) along the purple line yields two diffusion profiles, that in region I and that in region II, corresponding to the same time *t* into the coloration process. Figure 4b shows absorbance curves *A*_*t*_(*x*) derived using eq 3 for regions I and II. Now, the variable *x* denotes the distance away from the edge of the PMMA gap defining the onset of the corresponding region. The graph depicts three sets of corresponding absorbance curves recorded at the times *t* = 602, 2801, and 5201 s. As pointed out above, the absorbance is proportional to the hydrogen concentration in the WO_3_ thin film according to Lambert–Beer’s law. As a consequence, the absorbance profiles correspond to the spatial diffusion profiles at each recording time *t*. This allows us to directly compare the diffusion behavior in both regions: The diffusion in region I where the WO_3_ thin film is covered by NiO (blue curves) is faster than in region II where the WO_3_ thin film is covered by PMMA only (purple curves) at all times studied. After about 10 min of intercalation, the diffusion front on the WO_3_/NiO side (region I) is located at a distance of *x*_I_ = 40 μm in contrast to *x*_II_ = 30 μm on the WO_3_ side (region II). After 45 min the difference gets even bigger with *x*_I_ = 87 μm and *x*_II_ = 72 μm. After 85 min the difference is less pronounced but still fits the picture with *x*_I_ = 118 μm and *x*_II_ = 108 μm for the front in regions I and II, respectively. Thus, the diffusion front propagates faster in the WO_3_ layer covered by NiO than in the WO_3_ layer covered by PMMA. Furthermore, we find that the edge of the diffusion profile on the WO_3_ side (region II) is steeper than on the WO_3_/NiO side (region I) and the hydrogen concentration appears to increase faster and to reach higher values.

The absorbance profiles *A*_*t*_(*x*) in both regions reveal kinks at a specific absorbance value. The kinks have been shown to be related to the structural phase transition from the monoclinic/orthorhombic to the tetragonal phase taking place in the WO_3_ layer with increasing hydrogen concentration. The phase transition into the higher symmetry phase yields faster diffusion.^16^ It appears that the threshold absorbance value in region I where the WO_3_ is covered with NiO is somewhat lower than in region II.

Figure 4c shows a comparison of absorbance–time plots *A*_*x*_(*t*) recorded at three different distances *x* = 60, 100, and 120 μm from the corresponding edge of PMMA gap during the coloration experiment in the two regions. Again, the curves for WO_3_/NiO (region I) are shown in blue and those WO_3_ only (region II) in purple. Direct comparison of the three pairs of curves confirms that for all three distances *x* the diffusion front in region I covers the same distance faster than in region II. Furthermore, the absorbance in region I and thus the hydrogen concentration in WO_3_/NiO increases slower with time *t* than in WO_3_ covered solely by PMMA in region II. Eventually, after some time, the absorbance in region II for a given *x* overtakes that in region I, as already seen in Figure 4b. For all three different distances *x* from the PMMA gap, the diffusion front reaches the given distance about 10 min earlier in WO_3_/NiO (region I) than in solely WO_3_ (region II). Not surprising, the *A*_*x*_(*t*) plots in both regions show an initial rapid increase and subsequent flattening with increasing time *t*. This behavior also corresponds to the phase transition from the monoclinic/orthorhombic to the tetragonal phase taking place in the WO_3_ as demonstrated in our previous work.^16^

The main findings of the analysis of the data presented in Figure 3 are as follows: (i) hydrogen diffusion is faster when the WO_3_ is covered by NiO; (ii) the saturation value of the absorbance is lower in region I, where WO_3_ is covered by NiO than in region II where WO_3_ is covered by PMMA only (this difference is significant up to Δ*A* = 0.4); (iii) the characteristic diffusion profiles *A*_*x*_(*t*) and *A*_*t*_(*x*) in both regions reflect the concentration dependence of the hydrogen diffusion coefficient on the structural phase transition taking place in the WO_3_ layer with increasing hydrogen content, but the absorbance threshold is lower in the region where the WO_3_ is covered by NiO.

The observed findings can consistently be explained by the formation of a p–n heterojunction between NiO and WO_3_ and a corresponding positively charged depletion region in WO_3_. The lateral hydrogen diffusion in our multilayer sample solely takes place in the WO_3_ layer. Hydrogen diffusion cannot take place in the positively charged depletion region inside the WO_3_ layer as there are no electrons available to form the neutral hydrogen which actually diffuses inside the solid via hopping. This becomes more obvious, if we look at the following expression for the diffusion coefficient for hydrogen:^51^

4 $$D_{H}=\frac{1}{4F^{2}}\frac{\sigma_{e^{-}}\sigma_{H^{+}}}{\sigma_{e^{-}}+\sigma_{H^{+}}}\frac{\partial \mu_{H}}{\partial c_{H}}\propto \frac{\sigma_{e^{-}}\sigma_{H^{+}}}{\sigma_{e^{-}}+\sigma_{H^{+}}}$$

From the term describing the harmonic mean of ionic and electric conductivity, we can derive that the diffusion is governed by the ionic conductivity of H^+^ in bulk WO_3_, since σ_e^–^_ ≫ σ_H^+^_. However, as soon as we enter the depletion region, σ_e^–^_ becomes effectively zero and thus *D*_H_ = 0. Nonetheless, we believe that small amounts of hydrogen can still enter the depletion region as neutral hydrogen after having trapped an electron via

5 $$H⇌H^{+}+e^{-}$$

making hydrogen transport possible, even if the electronic conductivity is almost zero.^51^

The impact is 2-fold. First, hydrogen cannot enter the NiO layer and, second, the effective layer thickness of the WO_3_ in which lateral diffusion takes place is reduced. Figure 5a shows a schematic drawing of the sample with the depletion region and the resulting effective reduction of layer thickness. According to eq 3, the reduced effective layer thickness in WO_3_ covered by NiO (region I) also implies that less light is absorbed for a constant hydrogen concentration *y* than in the same WO_3_ layer covered solely by PMMA (region II). As a consequence, the maximum absorbance reached in region I is lower than in region II.

**Figure 5:**
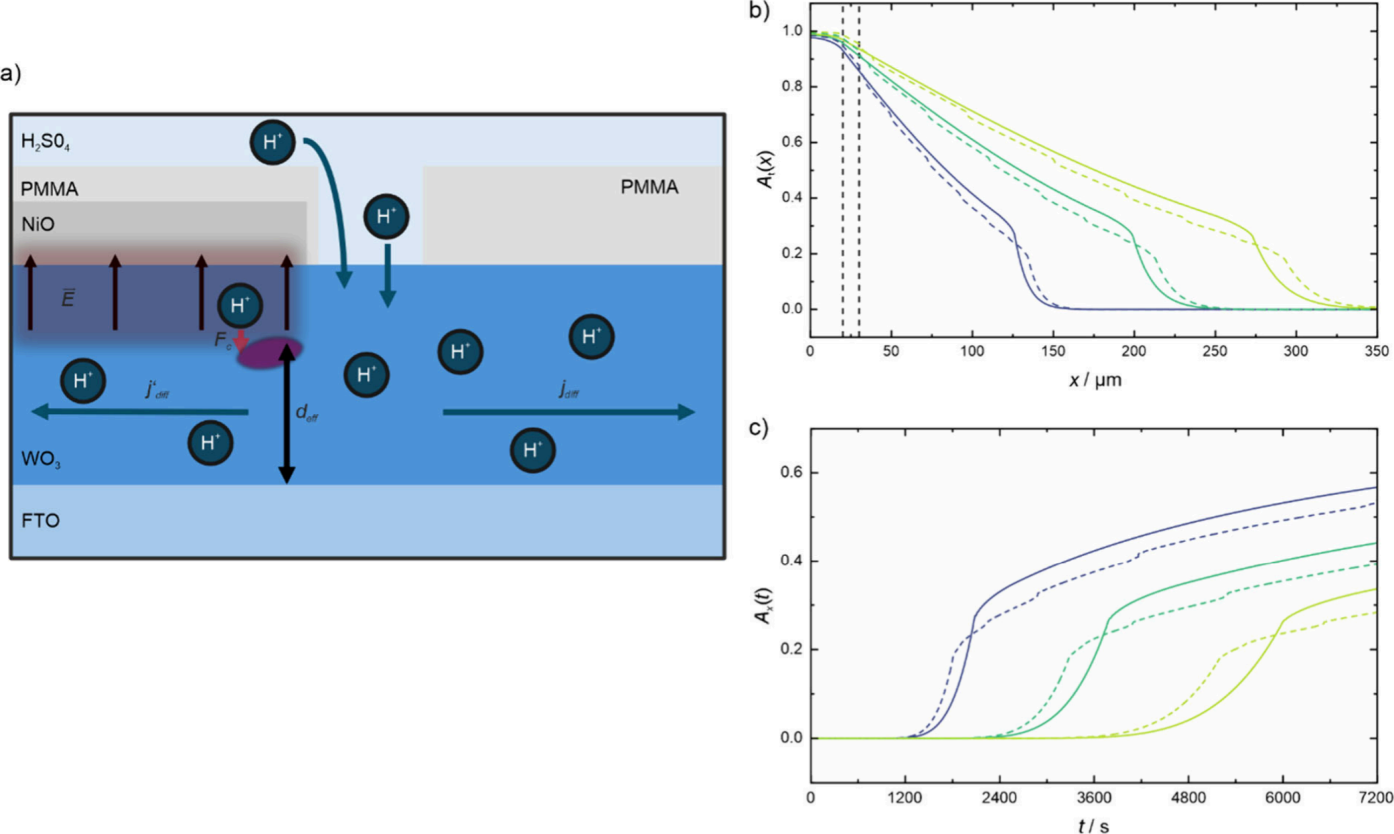
(a) Schematic cross-section of the sample during the diffusion process to explain the different diffusion behaviors caused by the electrical field/p–n heterojunction. Simulation of a diffusion without electrical field (solid lines) and with an electrical field taken account for (dashed lines): absorbance–distance plot (b) and absorbance–time plot (c).

However, not only the lower maximum absorbance in the WO_3_/NiO can be explained by this model, but also the supposedly faster diffusion. The decisive point here is the interplay between the depletion region, its electric field and the concentration-dependent diffusion coefficient of the polycrystalline WO_3_ layer. The electric field in the positively charged depletion region prevents the hydrogen from diffusing from the WO_3_ layer into the NiO. However, it does not completely prevent some ions from diffusing into this depletion region until an equilibrium of the particle flow by diffusion and the field-driven particle flow has been established. This results in an increased hydrogen concentration in the vicinity of the space charge region, which accelerates the aforementioned crystalline phase transition induced by the hydrogen concentration toward the tetragonal phase. This phase transition also increases the diffusion coefficient by approximately 1 order of magnitude, thereby favoring the macroscopically observed faster diffusion in the WO_3_ layer covered by the NiO.

### 3.3 Comparison with Simulation

In order to substantiate this hypothesis, a series of simulations of the diffusion process in our layered sample structure were conducted. The model accounts for the influence of the positively charged depletion region on hydrogen diffusion. The outcome of these simulations is presented in Figure 5b,c. Figure 5b illustrates the diffusion profiles, while Figure 5c shows the absorbance–time diagrams for varying distances from the edge of the PMMA gap. The shape of the simulated curves can thus be compared with the measurement data from Figure 4c,b, respectively. The dashed lines represent the results of the simulation that takes the depletion region into account, i.e., corresponding to WO_3_ covered by NiO (region I), and the solid lines represent the data from the simulation that does not include the space charge zone, i.e., corresponding to WO_3_ solely covered by PMMA (region II) in our experiment. It is evident from the simulated results of the absorbance profiles *A*_*t*_(*x*) that the diffusion front in the NiO/WO_3_ layer exhibits a propensity to diffuse deeper into the layer in comparison to the diffusion behavior observed in the pure WO_3_ layer. This phenomenon is further accentuated by the observation that the edge of the diffusion profile *A*_*t*_(*x*) is less pronounced, or the kink, which is attributed to the phase/concentration-dependent diffusion coefficient, manifests itself at a marginally lower absorbance threshold. It occurs at *A* ≈ 0.2 in case of WO_3_ with NiO on top and at about *A* ≈ 0.3 for pristine WO_3_ covered solely by PMMA. The measurement data is further corroborated by the simulated absorbance–time diagrams *A*_*x*_(*t*), which demonstrate a congruent process. The diffusion front reaches a given distance in WO_3_ covered by NiO faster than in WO_3_ covered solely by PMMA. In accordance with experiment, after the diffusion front has reached at a specific *x*, the simulated hydrogen concentration rises faster in WO_3_ solely covered by PMMA and eventually reaches higher values than in WO_3_ below NiO. These simulations thus fully support our explanation that the depletion region and the electric fields due to the p–n heterojunction between NiO and WO_3_ cause the diffusion in the WO_3_ layer below NiO to take place faster than in WO_3_ solely covered by PMMA. The dependence of the diffusion coefficient on hydrogen concentration inside the WO_3_ plays a decisive role in this context.

## 4 Conclusion

This study explores the lateral diffusion in WO_3_ thin films with a NiO top layer, focusing on the influence of the depletion region formed within the WO_3_ layer due to the presence of a p–n heterojunction at the NiO/WO_3_ interface. This heterojunction results in a depletion region that spans over 40% of the WO_3_ layer thickness, impacting the diffusion process in three significant ways. First, it serves as a barrier to the diffusion of hydrogen from the WO_3_ layer into the NiO layer. Second, the electric field within the depletion region reduces the effective thickness in which diffusion can occur in the WO_3_. Furthermore, the diffusion front’s velocity in in the WO_3_ layer plane below the NiO is increased, a phenomenon linked to the interaction between the depletion region and the concentration-dependent diffusion coefficient of hydrogen in polycrystalline WO_3_ thin films. This study demonstrates that by carefully incorporating semiconductor elements and applying external voltages, one can tailor the diffusion properties in thin films to specific requirements without altering the film itself. So by adjusting the applied voltage it is possible to alter the way how electrochromic devices change their optical properties, which could lead to advanced applications with layered structures. Our measurements serve as a proof of principle, illustrating the potential and universality of the proposed approach. Using a NiO/WO_3_ layer structure as a model system, we show that it is possible to investigate the diffusion of ions along a well-defined interface and gain fundamental knowledge on its effect. This opens up room for further investigations in the class of heterostructures consisting of a WO_3_ layer and a cover layer diving into studies of the impact of band alignment, doping and interface formation on the ion transport in the WO_3_ layer.

## Supplementary Material



## Notes

Surface morphology, crystal structure, depth profile surface roughness, bandgap/valence band offset, elemental mapping of sample cross-section, and calculation of refractive index (PDF)
