# Supplementary material for: Tailoring the Hydrogen Diffusion in Polycrystalline WO3 Thin Films by a p–n Heterojunction
Source: J Phys Chem C Nanomater Interfaces. 2025 Oct 3;129(41):18676–84. doi: 10.1021/acs.jpcc.5c04166 (PMC12536499; doi:10.1021/acs.jpcc.5c04166)
Supplement: Supplementary file 1 [file jp5c04166_si_001.pdf]

Supporting Information

Tailoring the Hydrogen Diffusion in Polycrystalline WO_3 Thin Films by a p-n Heterojunction

Tim K. Hecker, Jan L. Dornseifer, Markus S. Friedrich, Martin Becker, and Peter J. Klar*

Institute of Experimental Physics I and Center for Materials Research, Justus Liebig University Giessen, Heinrich Buff Ring 16, 35392 Giessen, Germany

E-mail: Tim.Hecker@exp1.physik.uni-giessen.de

S1. Surface Morphology

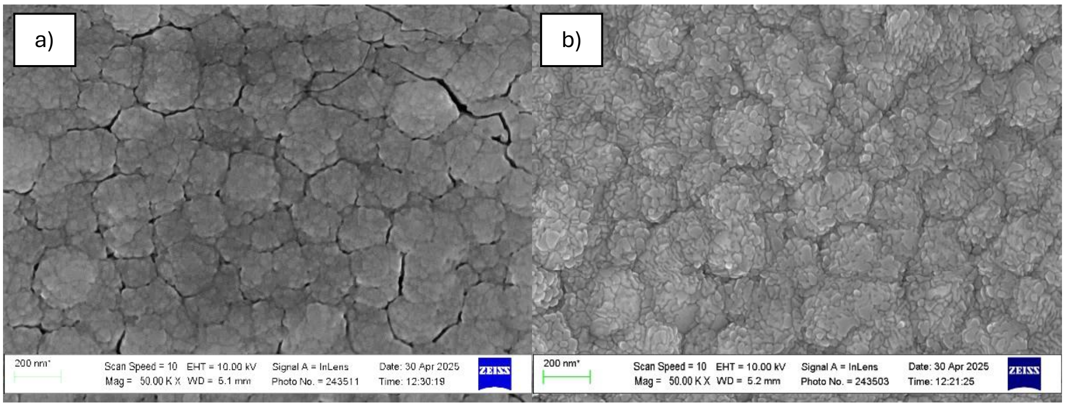

Figure S1. SEM images of the samples. a) annealed WO_3 film and b) WO_3 film with NiO top layer

Scanning electron microscope (SEM) images, as shown in Figure S1, give insight into the surface morphology of thin films. The annealed WO_3 thin film in Figure S1a consists of grains with sizes between 200 nm and 300 nm. Those grains are formed by even smaller crystallites. Due to annealing some cracks appear between some of the grains. After the sputter deposition of the NiO thin film on top of the WO_3, as seen in Figure S1b, the grain like structure of the underlying film is still discernable, but the whole surface is covered with small crystallites made of NiO, even covering all the cracks.

S2. Crystal Structure

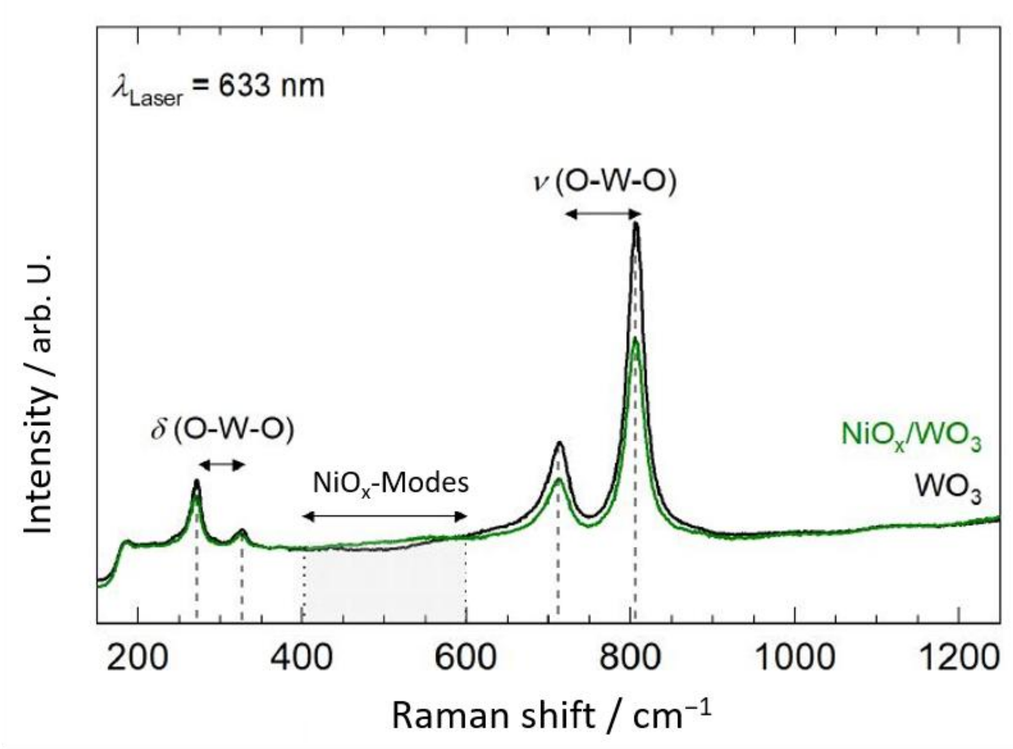

Figure S2. Raman spectra of the annealed WO_3 thin film (black) and the sample with the NiO top layer on the WO_3 film (green) measured with a 633 nm laser.

Raman investigations show the typical Raman modes for monoclinic/orthorhombic WO_3 at wavenumbers 713 cm^{-1} and 807 cm^{-1} and also the corresponding modes in the low frequency range.[1] The sample with the NiO layer on top of the WO_3 layer still shows all the features seen in the pristine film, but with lower intensity as before. In addition, broad modes of the NiO in the range between 400 cm^{-1} and 600 cm^{-1} ae detected.[2,3] This means that the underlying WO_3 film is not altered with respect to its crystal structure during the deposition of the top layer.

S3. Crystal Structure

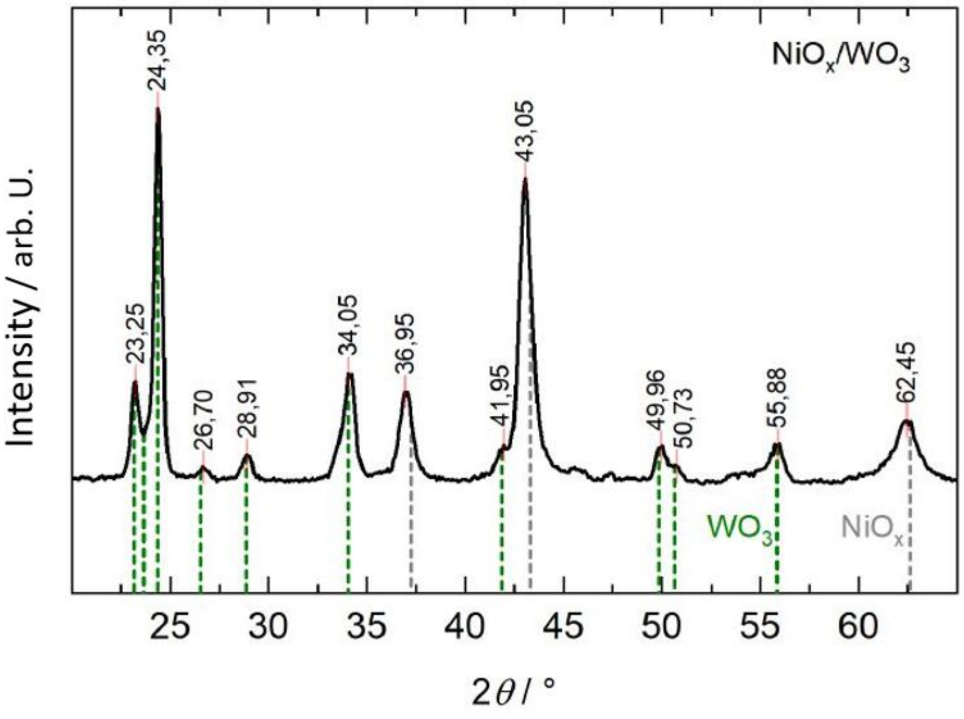

Figure S3. Diffraction pattern of a WO_3 thin film with NiO top layer. Dashed lines indicate the reflection angles of ideal WO_3 (green) and NiO (grey)

Results of the X-ray diffraction (XRD) investigation of the NiO/WO_3 layered structure are presented in Figure S3. The 2θ–XRD traces show reflexes of both thin films and reveal that both layers are polycrystalline. Furthermore, the comparison with a the XRD trace of the WO_3 film recorded prior to the deposition of NiO shows that, in the NiO/WO_3 layered structure, the underlying WO_3 film undergoes no changes due to deposition of the NiO top layer. Three characteristic reflections at reflection angles of $2\theta = 36.95°$, $2\theta = 43.05°$, and $2\theta = 62.45°$ that correlate to the (111), (200), and (220) plane, respectively, can be attributed to NiO.[4] Reflections at reflection angles of $2\theta = 23.25°$, $2\theta = 24.35°$, $2\theta = 26.70°$, $2\theta = 28.91°$, $2\theta = 34.05°$, $2\theta = 41.95°$, $2\theta = 49.96°$, $2\theta = 50.73°$ and $2\theta = 55.88°$ can be attributed to the WO_3 phases.[1,5,6]

S4. Depth Profile

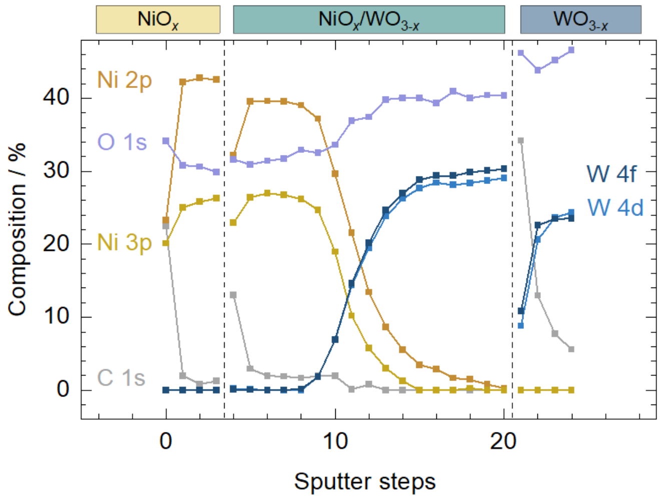

Figure S4. XPS depth profile of a WO_3 thin film with a NiO top layer.

Furthermore, an X-ray photoelectron spectroscopy (XPS) depth profile is displayed in Figure S4. The results corroborate that the material system consists of two well-defined layers of NiO and WO_3 with a rather narrow interface region where both NiO and WO_3 are present. The XPS depth profile reveals that the presence of tungsten is not detected within the NiO top layer during the initial sputter steps. However, as the measurement depth reaches a specific threshold, the presence of tungsten becomes noticeable. Subsequently with increasing number of sputter steps a pronounced increase in tungsten content is observed. This increase is accompanied by a reduction of the nickel content. After approximately 42 nm or 10 sputter steps, no nickel is detected anymore, indicating that the interface between the two layers is well-defined. Thus, the interface disorder is rather small and comparable to the roughness of the pristine WO_3 film.

S5. Surface Roughness

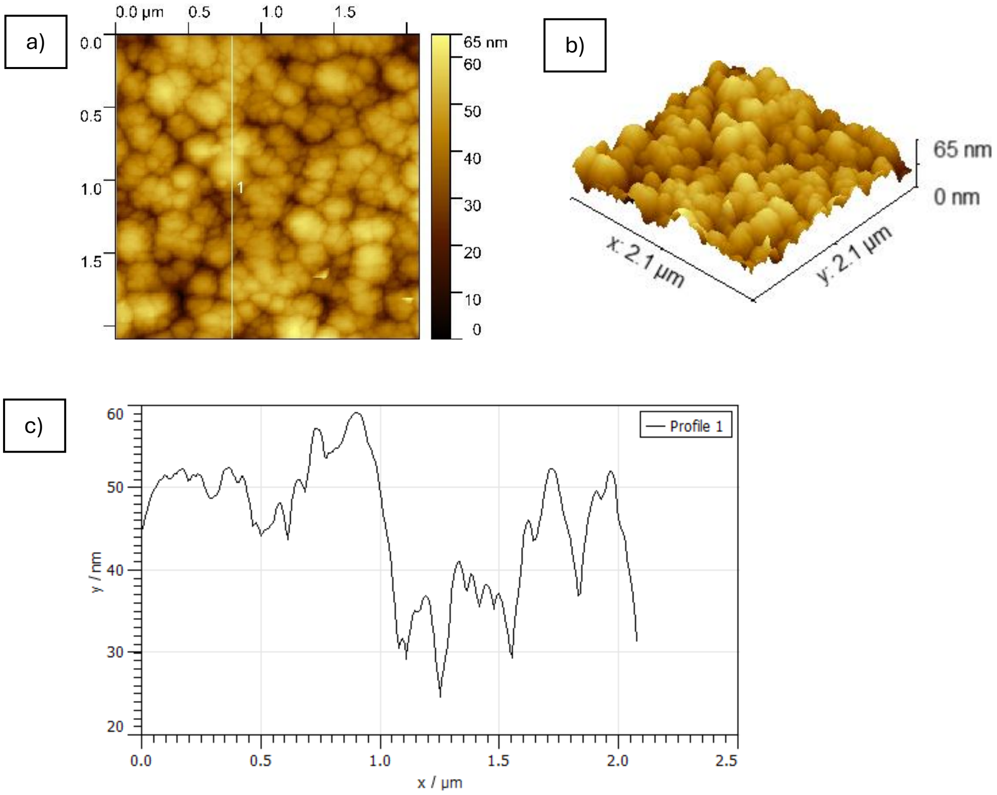

Figure S5. 2D and 3D representation of the surface morphology obtained by AFM imaging in a) and b) respectively. A 1D height representation along the straight line (indicated in a)) across the sample surface is given in c).

Results of Atomic Force Microscopy (AFM) imaging as depicted in Figure S5 underline that the width of the interface obtained by XPS profiling is in accordance with the surface roughness of the pristine WO_3 film. A surface roughness of the order of 30 nm are in line with the interface width or roughness of about 40 nm obtained by XPS.

S6. Bandgap/Valence Band Offset

In addition to providing insight into the distribution of elements within the thin film, the XPS measurements allow for a determination of the valence band offset (VBO) between NiO and WO_3. A value of $\Delta E_{VB} = 1.0$ eV is obtained using the following formula:[7]

$$VBO = (E^{B}_{CL2} - E^{B}_{VB}) - (E^{A}_{CL1} - E^{A}_{VB}) - (E^{I}_{CL2} - E^{I}_{CL1}) = \Delta E_{VB} \qquad \text{(S1)}$$

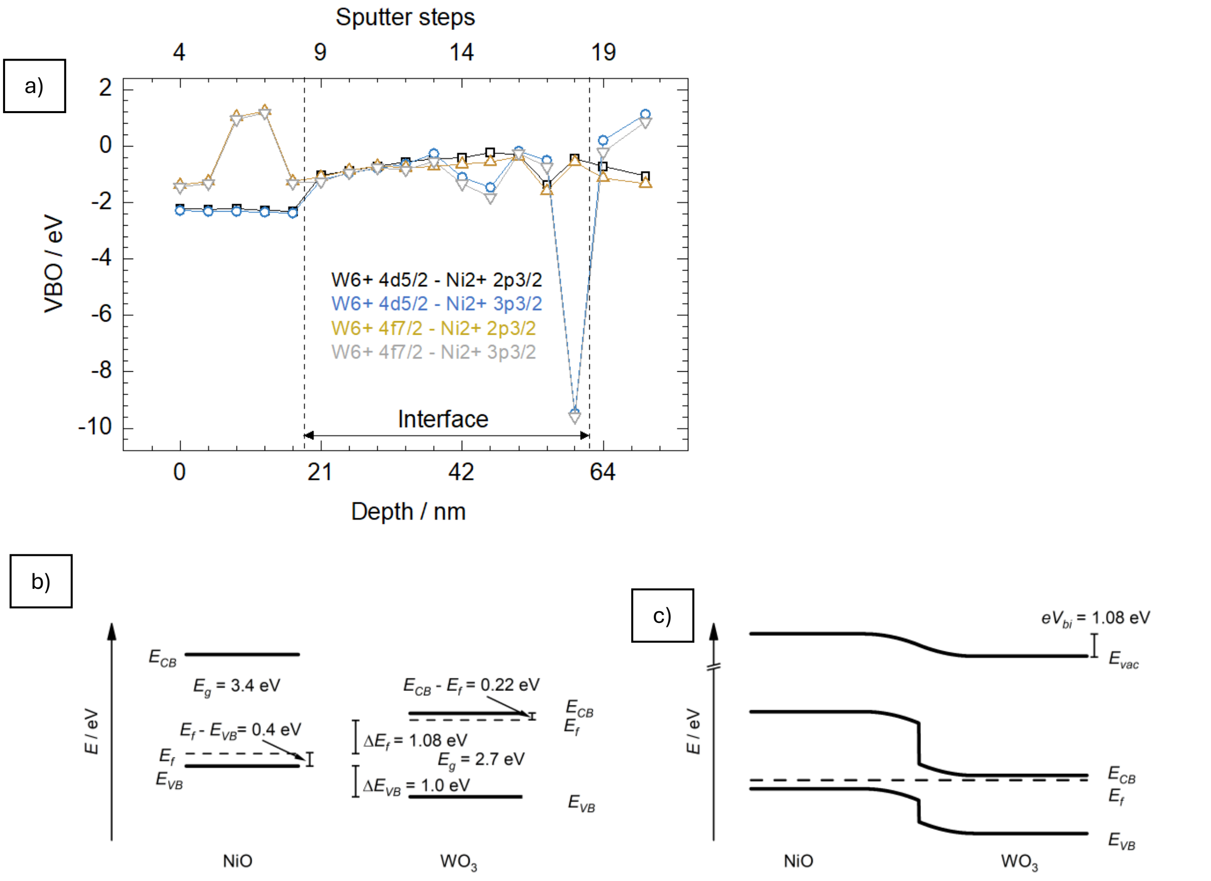

Figure S6. Graph a) calculated valence band offsets for all combinations of core levels. b) band diagram of the two materials with valence and conduction band and the Fermi energy with respect to the bands. c) band diagram of the two materials in contact, when an interface and depletion regions are formed

The built-in potential of the p-n heterojunction can then be calculated using the valence band offset from our XPS measurement and the bandgaps with relative positions of the Fermi energy to the bands (Figure 6a and b).

$$\Delta E_f = E - \Delta E_{VB} - \left(E_{f,NiO} - E_{VB,NiO}\right) - \left(E_{CB,WO_3} - E_{f,WO_3}\right) = 1.08 \text{ eV} \qquad \text{(S2)}$$

This leads to a build in potential of V_{bi}=1.08 V.[8–12]

S7. Elemental Distribution

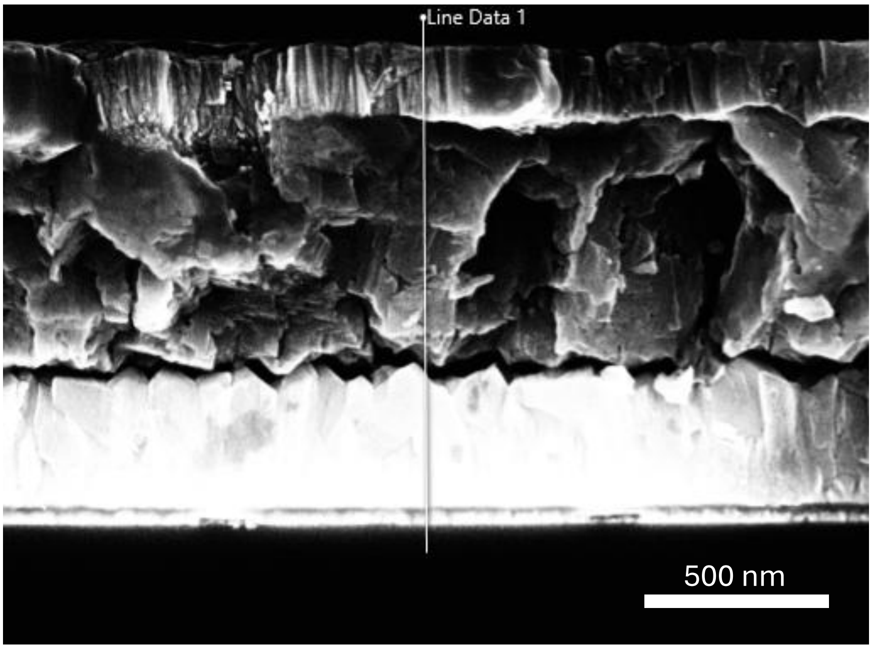

Figure S7. SEM image of the sample cross section with the position of the line scan indicated by a vertical line.

To further support the findings from the XPS depth profile we also conducted energy dispersive X-ray spectroscopy (EDX) on the cross section of the sample. The related SEM image is shown in Figure S7 also depicting the position of the line scan where we performed the EDX analysis.

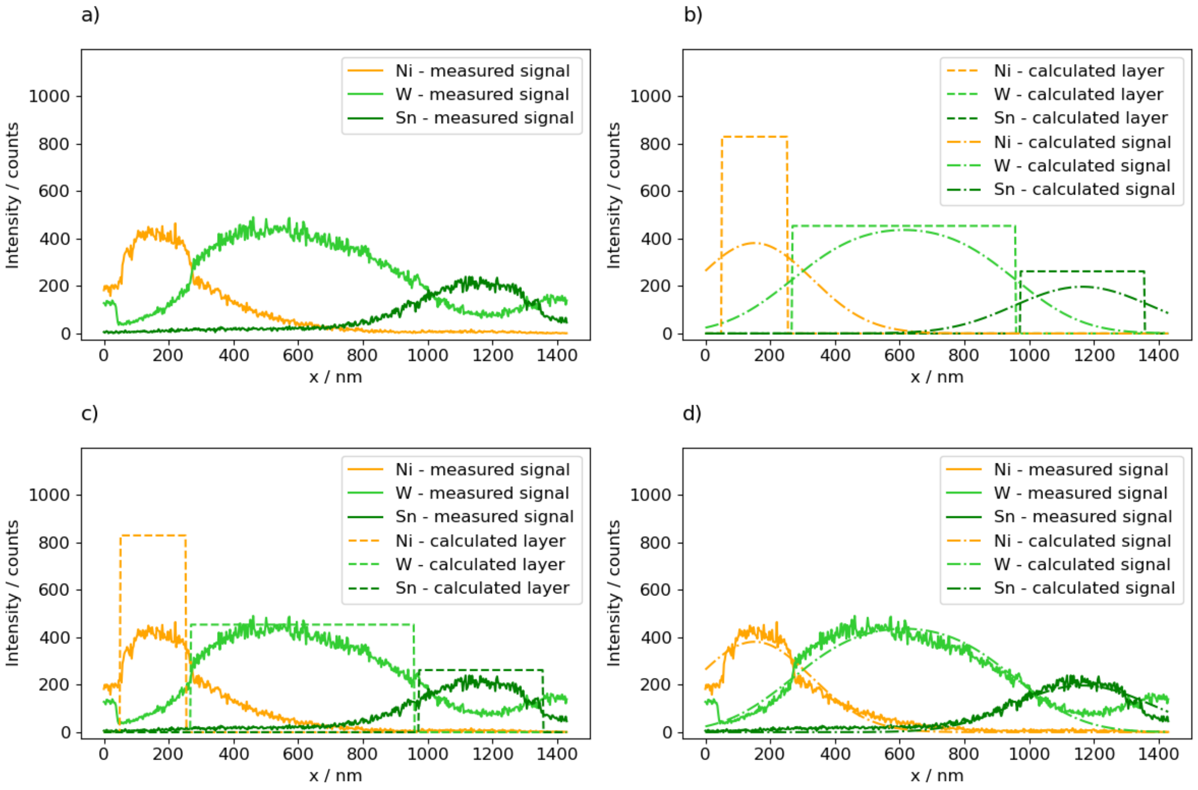

Figure S8. a) EDX signal for the elements Ni, W and Sn along a vertical line across the interfaces of the NiO/WO_3/FTO layers of the sample, b) assumed boxcar-like elemental distributions and resulting calculated EDX signals assuming a Gaussian for convolution, c) assumed boxcar-like elemental distributions in comparison to the measured EDX signal and d) calculated EDX signal in comparison to the measured EDX signal

Figure S8a shows the measured signals of the elements nickel (Ni), tungsten (W) and tin (Sn) that are used to identify the different layers. Since the spatial resolution of EDX is limited/hindered by the width of the interaction volume of the electrons from the electron beam with the analyzed material, the signals of the different layers fade into each other and overlap. The width of the interaction volume can be estimated using the Kanaya-Okayama equation[13]:

$$R = \frac{2.76\cdot10^{-11}\cdot A E_0^{\frac{5}{3}}}{\rho Z^{\frac{8}{9}}} \cdot \frac{(1+0.978\cdot10^{-6}\cdot E_0)^{\frac{5}{3}}}{(1+1.957\cdot10^{-6}\cdot E_0)^{\frac{4}{3}}} \qquad \text{(S3)}$$

where R is the maximum range of the electrons in the material, A the atomic weight, E_0 the kinetic energy of the electrons, ρ the density of the material and Z the atomic number. This leads to a maximum range of the electrons between R = 0.8 µm to R = 1 µm in the different

materials of the layer structure under investigation. Based on these estimated maximum ranges, it is reasonable to assume a half width of the interaction volume of about 0.5 µm.

Mathematically the measured signals can be understood as a convolution of the elemental distribution and a function that takes the extension of the interaction volume into account. For this function it is within reason to use a Gaussian distribution with a $3\sigma = 0.5$ µm, the previously mentioned half width of the interaction volume. When we now describe the elemental distribution of the elements as boxcar functions

$$\mathrm{boxcar}(x) = A(H(x-a) - H(x-b)) \qquad \text{(S4)}$$

with H(x) being the Heaviside function, we can adjust the parameters such, that the convolution of the boxcar and the Gaussian fits the measured signal best.

Figure S8b shows the corresponding calculated boxcar functions describing the three different layers and the calculated EDX signals from those along the EDX line scan. Figures 8c and 8d show the measured EDX signals in comparison to the boxcar profiles of the layers and calculated EDX signals; respectively.

These results are in good agreement with both, the SEM image of the cross section and the XPS depth profile, further and consistently confirming that the degree of intermixing between layers is low and the interfaces between the different layers are well defined.

S8. Calculation Refractive Index

Refractive Indices:

$n_{PMMA} = 1.5$[14]

$n_{WO3,pristine} = 2.2$[15]

$n_{WO3,intercalated} = 1.2$[15]

$n_{NiO} = 2.0$[16]

Formula reflectance:

$$R = \left(\frac{n_1 - n_2}{n_1 + n_2}\right)^2 \quad \text{(S5)}$$

PMMA/WO_3 interface (WO_3 side):

Bleached:

$$R_{bleached} = \left(\frac{n_{PMMA} - n_{WO_3,pristine}}{n_{PMMA} + n_{WO_3,pristine}}\right)^2 = \left(\frac{1.5-2.2}{1.5+2.2}\right)^2 = 0.0358 \quad \text{(S6)}$$

Intercalated:

$$R_{intercalated} = \left(\frac{n_{PMMA} - n_{WO_3,intercalated}}{n_{PMMA} + n_{WO_3,intercalated}}\right)^2 = \left(\frac{1.5-1.2}{1.5+1.2}\right)^2 = 0.0123 \quad \text{(S7)}$$

WO_3/NiO interface (WO_3/NiO side):

Bleached:

$$R_{bleached} = \left(\frac{n_{NiO} - n_{WO_3,pristine}}{n_{NiO} + n_{WO_3,pristine}}\right)^2 = \left(\frac{2.0-2.2}{2.0+2.2}\right)^2 = 0.0023 \quad \text{(S8)}$$

Intercalated:

$$R_{intercalated} = \left(\frac{n_{NiO} - n_{WO_3,intercalated}}{n_{NiO} + n_{WO_3,intercalated}}\right)^2 = \left(\frac{2.0-1.2}{2.0+1.2}\right)^2 = 0.0625 \quad \text{(S9)}$$

Calculation of change in absorbance ΔA:

We can calculate the intensity of the transmitted light I with respect to the change in refractive indices as follows, with I_R as the intensity of the transmitted light neglecting such changes.

$$I = \left(\frac{1-R_{intercalated}}{1-R_{bleached}}\right) \cdot I_R \tag{S10}$$

The formula for the absorbance can be modified to

$$A = -\ln\left(\frac{I}{I_0}\right) = -\ln\left(\frac{\left(\frac{1-R_{intercalated}}{1-R_{bleached}}\right) \cdot I_R}{I_0}\right) = -\ln\left(\frac{I_R}{I_0}\right) - \ln\left(\frac{1-R_{intercalated}}{1-R_{bleached}}\right) \tag{S11}$$

With the change in absorbance due to changes in refractive indices ΔA given by

$$\Rightarrow \Delta A = \ln\left(\frac{1-R_{bleached}}{1-R_{intercalated}}\right) \tag{S12}$$

This yields ΔA = -0,0241 for the pure WO_3 side and ΔA = 0,0622 for the NiO/WO_3 side. So the maximum difference in measured absorbance due to changes in the refractive indices comparing the two sides is ΔA_{max} = 0,0863.

References

(1) Cazzanelli, E.; Vinegoni, C.; Mariotto, G.; Kuzmin A.; Purans, J. Raman Study of the Phase Transitions Sequence in Pure WO_3 at High Temp and in H_xWO_3. *Solid State Ion.* **1999**, *123*, 67–74. https://doi.org/10.1016/S0167-2738(99)00101-0.

(2) Dietz, R. E.; Parisot, G. I.; Meixner, A. E. Infrared Absorption and Raman Scattering by Two-Magnon Processes in NiO. *Phys. Rev. B* **1971**, *4* (7), 2302–2310. https://doi.org/10.1103/PhysRevB.4.2302.

(3) Mironova-Ulmane, N.; Kuzmin, A.; Steins, I.; Grabis, J.; Sildos, I.; Pärs, M. Raman Scattering in Nanosized Nickel Oxide NiO. *J. Phys. Conf. Ser.* **2007**, *93*, 012039. https://doi.org/10.1088/1742-6596/93/1/012039.

(4) Hotovy, I.; Huran, J.; Spiess, L. Characterization of Sputtered NiO Films Using XRD and AFM. *J. Mater. Sci.* **2004**, *39* (7), 2609–2612. https://doi.org/10.1023/B:JMSC.0000020040.77683.20.

(5) Kuzmin, A.; Purans, J.; Cazzanelli, E.; Vinegoni, C.; Mariotto, G. X-Ray Diffraction, Extended x-Ray Absorption Fine Structure and Raman Spectroscopy Studies of WO_3 Powders and $(1-x)WO_{3-y}\cdot xReO_2$ Mixtures. *J. Appl. Phys.* **1998**, *84* (10), 5515–5524. https://doi.org/10.1063/1.368596.

(6) Yebka, B. Electrochemical Li^+ Insertion in $WO_{3-x}TiO_2$ Mixed Oxides. *Solid State Ion.* **1997**, *104* (3–4), 169–175. https://doi.org/10.1016/S0167-2738(97)00439-6.

(7) Benz, S. L.; Becker, M.; Polity, A.; Chatterjee, S.; Klar, P. J. Determining the Band Alignment of Copper-Oxide Gallium-Oxide Heterostructures. *J. Appl. Phys.* **2021**, *129* (11), 115305. https://doi.org/10.1063/5.0036591.

(8) Egbo, K. O.; Shil, S. K.; Kwok, C. G.; Wang, Y.; Liu, C. P.; Yu, K. M. Band Alignment of Wide Bandgap NiO/MoO_3 and NiO/WO_3 p-n Heterojunctions Studied by High-Resolution X-Ray Photoelectron Spectroscopy. *J. Alloys Compd.* **2021**, *876*, 160136. https://doi.org/10.1016/j.jallcom.2021.160136.

(9) Makhado, K. P.; Mphahlele-Makgwane, M. M.; Kumar, N.; Baker, P. G. L.; Makgwane, P. R. Current Updates on P-Type Nickel Oxide (NiO) Based Photocatalysts towards Decontamination of Organic Pollutants from Wastewater. *Mater. Today Sustain.* **2024**, *25*, 100664. https://doi.org/10.1016/j.mtsust.2023.100664.

(10) Wu, P.; Liu, Z.; Chen, D.; Zhou, M.; Wei, J. Flake-like NiO/WO_3 p-n Heterojunction Photocathode for Photoelectrochemical Water Splitting. *Appl. Surf. Sci.* **2018**, *440*, 1101–1106. https://doi.org/10.1016/j.apsusc.2018.01.292.

(11) Hüfner, S.; Steiner, P.; Sander, I.; Reinert, F.; Schmitt, H.; Neumann, M.; Witzel, S. The Electronic Structure of NiO Investigated by Photoemission Spectroscopy. *Solid State Commun.* **1991**, *80* (10), 869–873. https://doi.org/10.1016/0038-1098(91)90523-X.

(12) Ma, Y.; Qin, Y.; Porter, M.; Spencer, J.; Du, Z.; Xiao, M.; Wang, B.; Wang, Y.; Jacobs, A. G.; Wang, H.; Tadjer, M.; Zhang, Y. Wide-Bandgap Nickel Oxide with Tunable Acceptor Concentration for Multidimensional Power Devices. *Adv. Electron. Mater.* **2023**, 2300662. https://doi.org/10.1002/aelm.202300662.

(13) K Kanaya; S Okayama. Penetration and Energy-Loss Theory of Electrons in Solid Targets. *J. Phys. Appl. Phys.* **1972**, *5* (1), 43–58. https://doi.org/10.1088/0022-3727/5/1/308.

(14) Michel, P.; Dugas, J.; Cariou, J. M.; Martin, L. Thermal Variations of Refractive Index of PMMA, Polystyrene, and Poly (4-Methyl-1 -Pentene). *J. Macromol. Sci. Part B* **1986**, *25* (4), 379–394. https://doi.org/10.1080/00222348608248046.

(15) Yuan, G.; Hua, C.; Huang, L.; Defranoux, C.; Basa, P.; Liu, Y.; Song, C.; Han, G. Optical Characterization of the Coloration Process in Electrochromic Amorphous and Crystalline WO_3 Films by Spectroscopic Ellipsometry. *Appl. Surf. Sci.* **2017**, *421*, 630–635. https://doi.org/10.1016/j.apsusc.2016.10.176.

(16) Valyukh, I.; Green, S.; Arwin, H.; Niklasson, G. A.; Wäckelgård, E.; Granqvist, C. G. Spectroscopic Ellipsometry Characterization of Electrochromic Tungsten Oxide and Nickel Oxide Thin Films Made by Sputter Deposition. *Sol. Energy Mater. Sol. Cells* **2010**, *94* (5), 724–732. https://doi.org/10.1016/j.solmat.2009.12.011.
